# Research on the impact of rotor wear on the effectiveness of the aluminium refining process

**DOI:** 10.1038/s41598-023-44562-0

**Published:** 2023-10-18

**Authors:** Ladislav Socha, Tomáš Prášil, Karel Gryc, Jana Svizelova, Mariola Saternus, Tomasz Merder, Jacek Pieprzyca, Petr Nuska

**Affiliations:** 1 Environmental Research Department, Institute of Technology and Business in České Budějovice, Okružní 517/10, 370 04 České Budějovice, Czech Republic; 2 University of West Bohemia, Univerzitní 2732, 301 00 Plzeň, Czech Republic; 3 MOTOR JIKOV Slévárna a.s., Die-casting Division, Kněžskodvorská 2277, 370 04 České Budějovice, Czech Republic; 4 Faculty of Materials Engineering, Silesian University of Technology, Krasinskiego 8, 40-019 Katowice, Poland

**Keywords:** Engineering, Materials science

## Abstract

Physical modelling is successfully used to understand mechanisms involved in the aluminium refining process by injecting inert gas into the liquid metal through rotors. Two types of industrial impellers, which are extremely different in construction, were tested in the research. The aim of the research was to determine the effectiveness of their operation depending on their degree of wear. This type of research has not been tested on water models so far. During the process, the parameters were changed, such as the gas flow rate from 13 to 19 L/min, the rotor speed from 325 to 400 rpm and the height of the rotor from the bottom of the refining reactor. Tests were carried out for new and worn rotors. Oxygen removal rate curves were prepared on the basis of tests determining changes in oxygen content in the model liquid as a function of time for changing rotor speed values. It was found that the efficiency of hydrogen removal from the model liquid was higher when worn impellers were used in the model. In order to verify results of model tests, a metallographic analysis of samples obtained in industrial conditions and using the analysed process parameters was carried out.

## Introduction

Recycling methods are today one of the dominant sources of aluminium and its alloys. In the technologies used for smelting aluminium scrap, the stage of refining the liquid metal plays an important role^1,2^. One commonly used technique is purging the metal bath with an inert gas, most often argon or nitrogen^3,4^. This is done to remove hydrogen; however, it also removes metallic and non-metallic impurities arising during the secondary aluminium smelting process^5,6^. A variant of this procedure is argon injection through the rotating impeller^7–9^. In this case, the basic process parameters regulating the refining process and determining its effectiveness are the rotor speed and the value of the gas stream^10,11^. Determining these parameters in industrial conditions is extremely difficult and expensive. Therefore, they are usually determined on the basis of model tests using physical water models and numerical simulations carried out simultaneously in order to verify the results^12–17^. The results of laboratory tests obtained in this way are highly reliable^18–20^.

**Figure 1:**
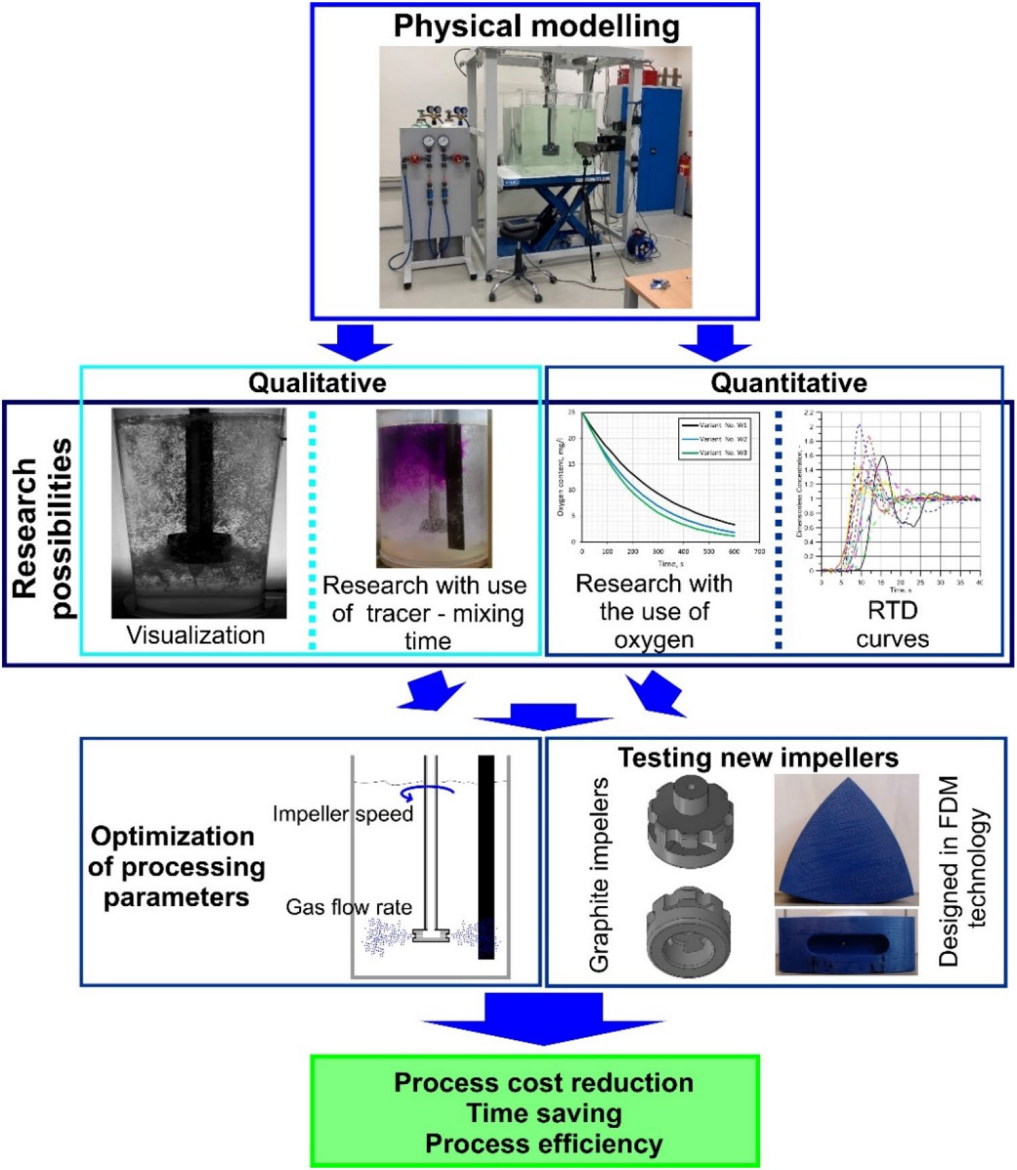
Schematic diagram of the types of research used in physical modelling.

Physical modelling is widely used to determine the hydrodynamic conditions of metallurgical reactors. Physical models are built according to specific rules based on fluid mechanics and dimensional analysis^21,22^. In general, measurement techniques used in physical modelling can be divided into techniques used for imaging the movement of particles, e.g. non-metallic inclusions or inert gas bubbles, and for imaging the movement of metal, e.g. metal alloy homogenization mechanisms and efficiency^23,24^. Research conducted on physical models can be divided into two groups depending on the type of results: qualitative and quantitative. As part of the qualitative tests, in the case of the considered process—Fig. 1—the movement of gas bubbles in the model liquid (most often water) simulating aluminium and its alloys is observed. Based on the dispersion patterns obtained (column flow, minimum, intimate, uniform or excessive dispersion)^25^, it is possible to assign a specific type of dispersion to specific process parameters, such as gas flow rate and rotor speed. If it is necessary to visualize the method of mixing and homogenizing aluminium or its alloys, a coloured marker is introduced into the model liquid^26^. This method is also widely used in studies of hydrodynamic phenomena occurring in various metallurgical reactors, both in non-ferrous metallurgy and steel metallurgy^27–29^. This method was successfully applied in the studies of aluminium refining published in^30^. As a result, it was possible to determine the dispersion time of gas bubbles in the liquid (mixing degree) and compare it with the results for the remaining rotors. The second group of research using water models is qualitative research. Most often it consists in determining the change in the concentration of the marker added to the model liquid as a function of time and determining the mixing characteristics called RTD curves (Residence Time Distribution)^31–35^. These studies have also been successfully applied in the aluminium refining process by blowing gas through a rotating impeller^36^. In this type of research, the degree of oxygen removal from the model liquid is measured as an analogy of hydrogen removal from aluminium. The model liquid is oxygenated to the degree of saturation, then argon is introduced into the solution and changes in oxygen concentration in the solution are measured^23,36^. In this way, the efficiency of the gas consumption can be calculated. The diagram in Fig. 1 shows that the model tests are aimed either at optimizing the process parameters or testing new rotors, both real, industrial, and those printed using Fused Deposition Modelling (FDM) 3D printing^36^. Therefore, model studies offer reduced costs when compared with industrial studies^37–39^. The practical application of the results allows for the optimal selection of process parameters for a given rotor, and consequently for reducing the time of the refining process and increasing its efficiency^40–42^.

The article emphasizes that the geometric structure of the rotor itself is also important for the proper course of the liquid aluminium refining process. Therefore, works on this subject are appearing more often in the professional literature^43–46^. However, they mainly concern the problem of the impact of the rotor structure on the mechanism of the formation of gas dispersion zones in the liquid and, consequently, on the course of the refining process. They do not take into account the phenomenon of rotor wear during smelting. Industrial impellers are made of various materials, most commonly silicon carbide or graphite, and have a specific service life. Their geometric parameters change over their period of use. Therefore, the course and efficiency of the process change as the impeller wears out. The mechanism of this problem has not yet been analysed. However, this is an important issue that affects both the refining of liquid aluminium itself and the economics of the entire production process. Therefore, this article is devoted to this issue and the main aim of the research was to verify (confirm) the noticed, unexpected phenomenon of improvement in the effectiveness of the liquid aluminium refining treatment during its duration and the progressive degree of rotor wear. The research used both qualitative physical modelling—visualization, and quantitative—measurements of oxygen removal from the model liquid.

## Research methodology

Physical modelling of technological processes is often identified with the experiment itself. In fact, the first phase of modelling is quite a theoretical matter, based on understanding the process under study. At this stage of the research, the most important parameters of the process are identified and the research problem should be defined. These activities allow the determination and selection of the appropriate method of building a physical model. In the case of the considered process, it was decided to build a physical model of the device for refining liquid aluminium on a scale of 1–1. The justification for choosing such a linear scale is the possibility of using real rotors with varying degrees of wear in the experiment. The principles of physical modelling described in many publications^21–23^, in addition to geometric similarity, i.e. maintaining the ratio of characteristic dimensions in the model to these dimensions in real conditions, also require maintaining dynamic and kinematic similarity. The dynamic similarity of the model to the real object was determined on the basis of the dimensional analysis of the Navier–Stokes equations using the Buckingham π theorem. In this way, the basic criteria of dynamic similarity were determined in the form of criterion numbers Fr (Froude), We (Weber) and Re (Reynolds). The equality of the values of these numbers calculated for the model and the real object is a necessary and sufficient condition to obtain full dynamic similarity between the model and the real object. However, satisfactory modelling results can also be obtained using the so-called incomplete dynamic similarity, in which the agreement of the values of one or more criterion numbers is met. In most cases, when considering single-phase flows, the equality of the Froude number is a sufficient criterion for similarity. In the analysed case, however, there is a phenomenon of gas flow through the liquid, i.e. multiphase flow. The Froude number then has to be modified due to differences in the density of liquid and gas. Finally, the dominant similarity criterion was the modified Froude number in the form:

1 $${\text{Fr}}_{{\text{N}}} = \frac{{\uprho _{{\text{g}}} \cdot {\text{v}}^{2} }}{{\uprho _{{\text{l}}} \cdot {\text{g}} \cdot {\text{L}}}}$$

where ρ_g_, gas density; ρ_l_, liquid density; g, acceleration due to gravity; L, liquid height in the model; v, gas injection velocity.

**Figure 2:**
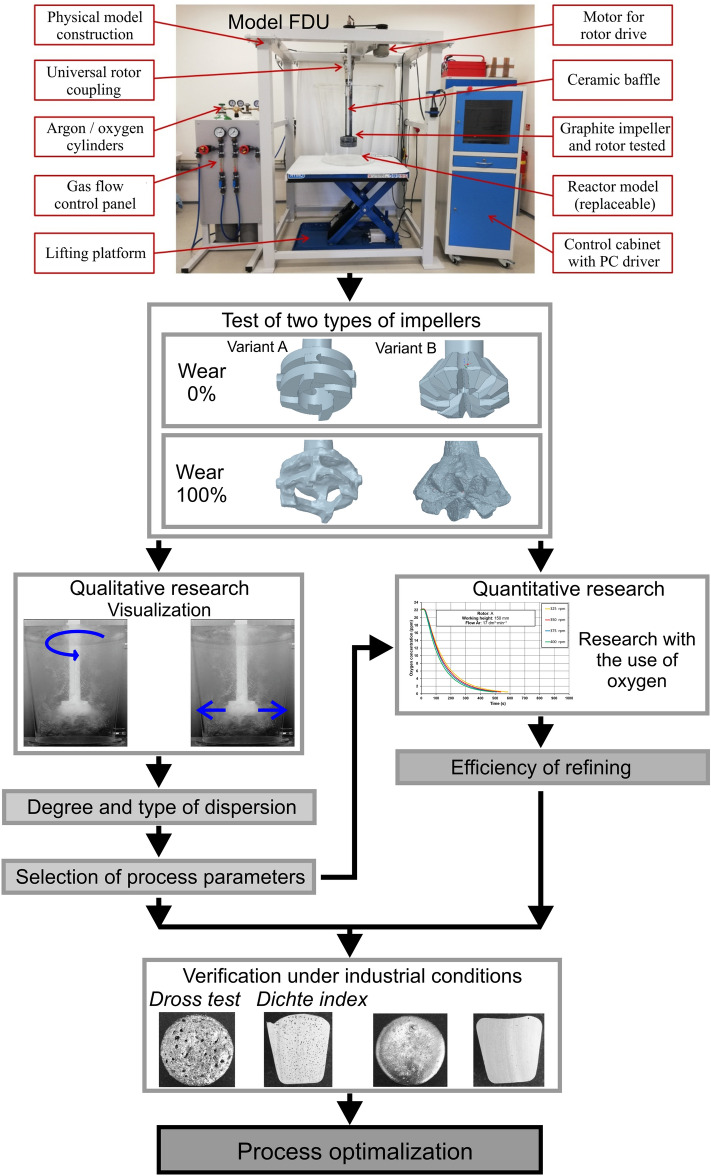
Diagram of the research methodology.

**Figure 3:**
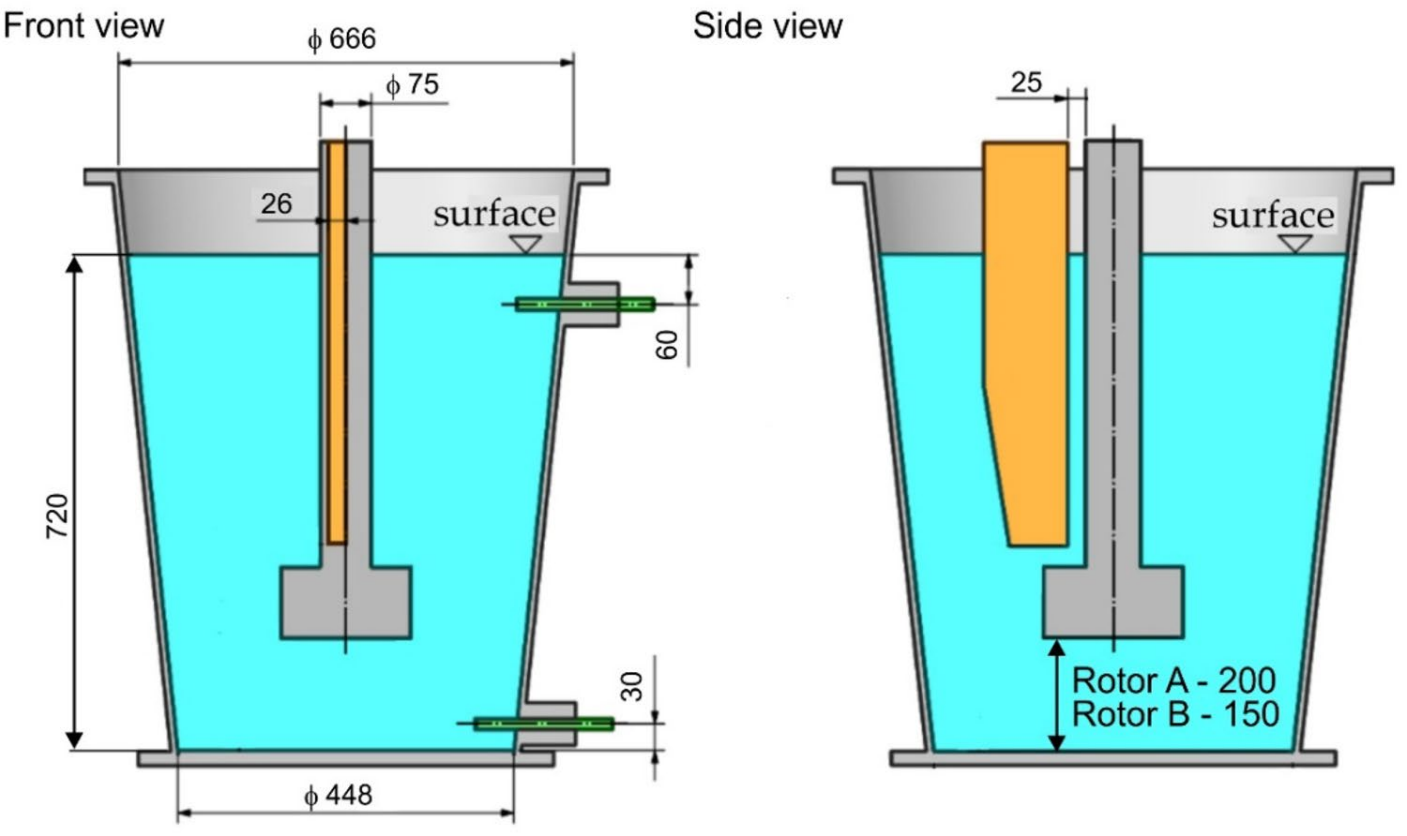
Basic dimensional data of the physical model and individual components.

The dynamic similarity determined in this way makes it possible to determine the kinematic similarity. The values of the technological parameters of the process are determined on the basis of kinematic similarity, i.e. the values of the stream blown into the gas model and the rotational speed of the rotor in the model. After this stage, the experiments began. Figure 2 shows a diagram of the research methodology. The physical model consisted of the main segment, i.e. the refining ladle, and auxiliary segments ensuring the required functionality and precision of controlling the assumed parameters of the experiment. These include hydraulic installations and control and measurement equipment. Figure 3 shows the diagram and basic dimensions of the refining ladle model as a segment of the main test stand shown in Fig. 2.

Two types of impellers differing in construction and used in industry, marked as variant A and B, were tested in the research. The aim of the research was to determine the efficiency of their work depending on their degree of wear. In order to take into account the degree of impeller wear, it was assumed in the test programme that a new impeller starting work at the liquid aluminium refining station will be marked as 0%, while the rotor which has finished work at the station will be marked as 100%. Figure 2 shows the types of impellers selected for testing before and after use at the aluminium refining station. The images were made using 3D scanning and then CAD engineering design tools.

Taking into account the actual course of the aluminium refining process in industrial conditions, a programme for the laboratory tests was developed—Table 1 presents the range of processing parameters to be tested (accuracy of measuring rotary impeller speed was ± 1 rpm and flow rate of refining gas ± 0.1 dm^3^ min^−1^). These tests had a twofold character: visualization of the formation and dispersion of gas bubbles in the volume of the model liquid, i.e. water, and the efficiency of oxygen removal from the model liquid under the influence of injected inert gas as an analogy of hydrogen removal from aluminium. For continuous measurement of the oxygen content in the simulant, optical probes (accuracy ± 1%) and METTLER-TOLEDO InPro6860i/12/120/mA Ex components were installed, enabling the measurement of oxygen concentration in the assumed range up to 26 mg L^−1^ (26 ppm).

**Table 1:** Parameters used in model tests—specific for rotor A and B.

Parameters, unit	Value															
Rotor A and B																
Rotary impeller speed, rpm	325				350				375				400			
Gas flow rate, dm^3^·min^−1^	13	15	17	19	13	15	17	19	13	15	17	19	13	15	17	19
Distance of the impeller from the bottom of the ladle, mm									Rotor A				Rotor B			
									200				150			

After carrying out the model tests and applying the results in industrial tests, samples were taken from the produced material for qualitative analysis using the Dross Test, where the Dichte Index was calculated. Based on these results, the refining process was optimized, which was the goal of the research programme.

## Research results

**Figure 4:**
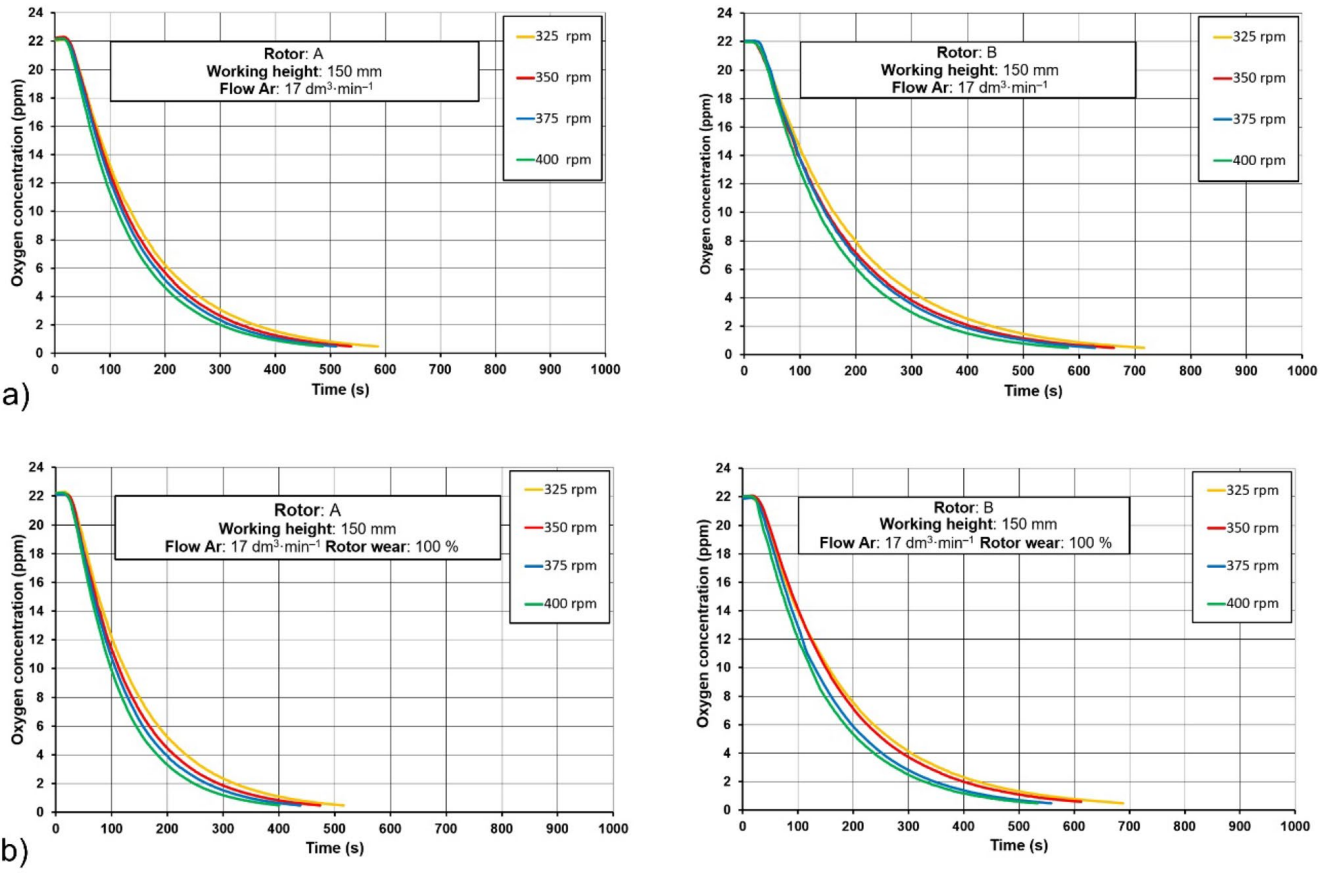
Oxygen removal curves for rotors A and B for (**a**) a new rotor, wear 0% and (**b**) a worn rotor, wear 100%.

On the basis of the tests for determining changes in the oxygen content in the model liquid as a function of time for individual values of the rotary impeller speed, curves of oxygen removal rate were prepared. Example graphs are shown in Fig. 4.

**Figure 5:**
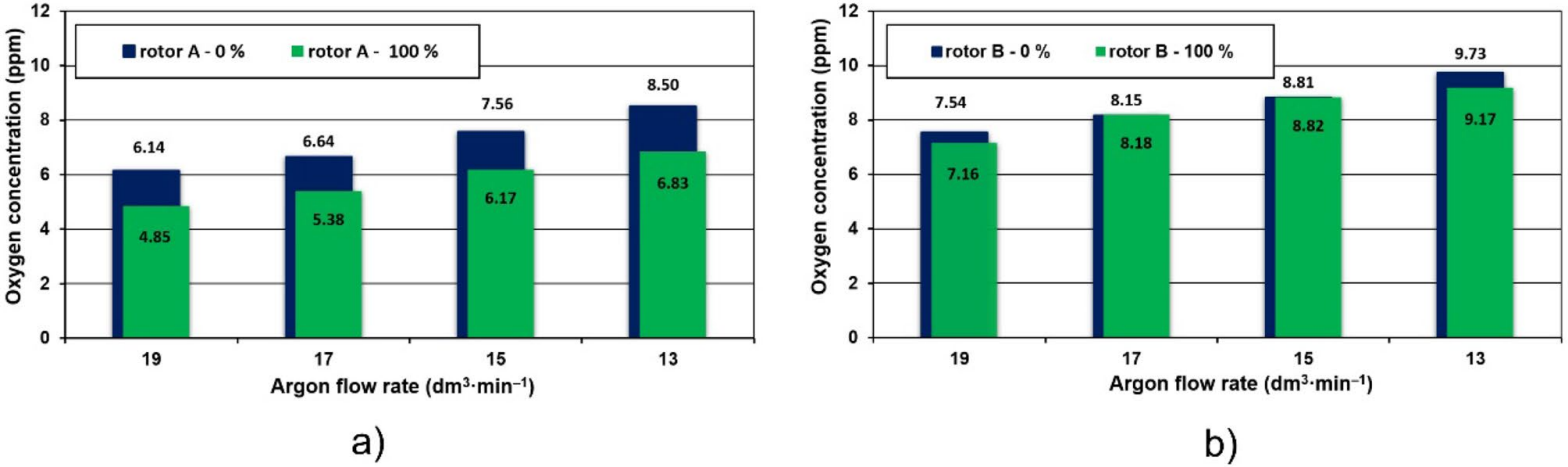
Averaged changes in oxygen concentration in the model liquid for individual values of the flow rate of the injected inert gas: (**a**) rotor A, (**b**) rotor B.

In all cases, the best results were obtained for rotor A. At the rotary impeller speed of 325 rpm, it can be seen that the effective time of oxygen removal is the longest and amounts to about 600 s for the new rotor, while in the case of the worn rotor it is reduced to 520 s. The values for rotor A were obtained for rotational speeds of 375 and 400 rpm and the worn rotor for about 400 s. The best values for rotor A were obtained for rotary speeds of 375 and 400 rpm and the worn rotor—about 400 s. These times for rotor B are much longer and amount to a rotary speed of 325 rpm for a new rotor 720 s and for a worn rotor 690 s, and for a rotary speed of 375 and 400 rpm for a new and worn rotor, approximately 580 s and 550 s, respectively. The analysis of the curves allowed for better efficiency of rotor A operation. The minimum times for lowering the oxygen concentration in the model liquid for all variants of the experiment are shorter. However, an unexpected and surprising phenomenon was also noticed. The efficiency of oxygen removal from the model liquid is higher when worn rotors are used in the model, which in turn may lead to the conclusion that with the elapse of time of the refining process, despite the wear of the rotors, the efficiency of the process does not decrease, but increases slightly. This trend is presented in Fig. 5 in the form of collective comparative charts prepared for rotors A and B, showing the average time of oxygen removal for individual values of the injected inert gas stream.

**Figure 6:**
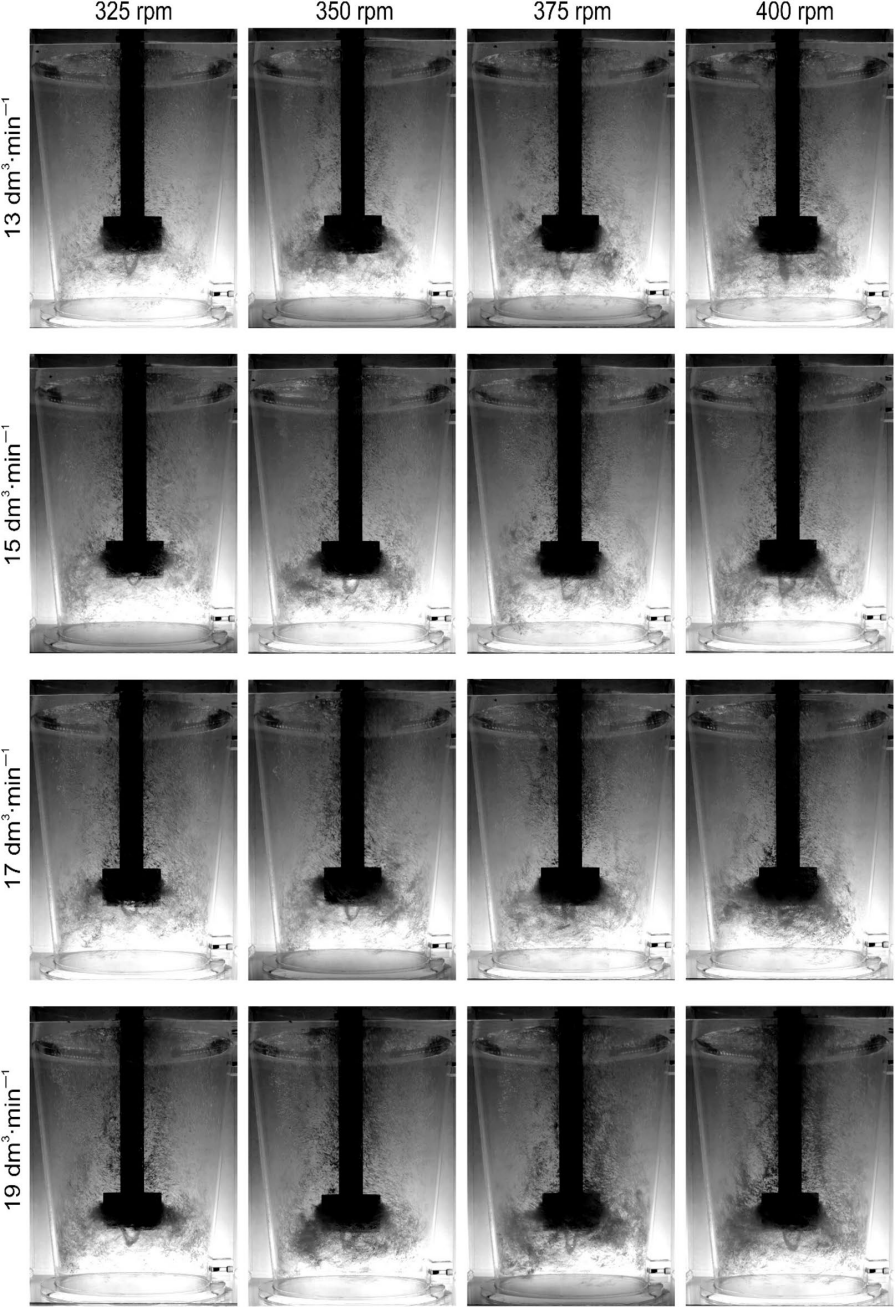
Demonstration of the nature of internal flows, behaviour and distribution of bubbles of injected argon—rotor A wear 0%.

**Figure 7:**
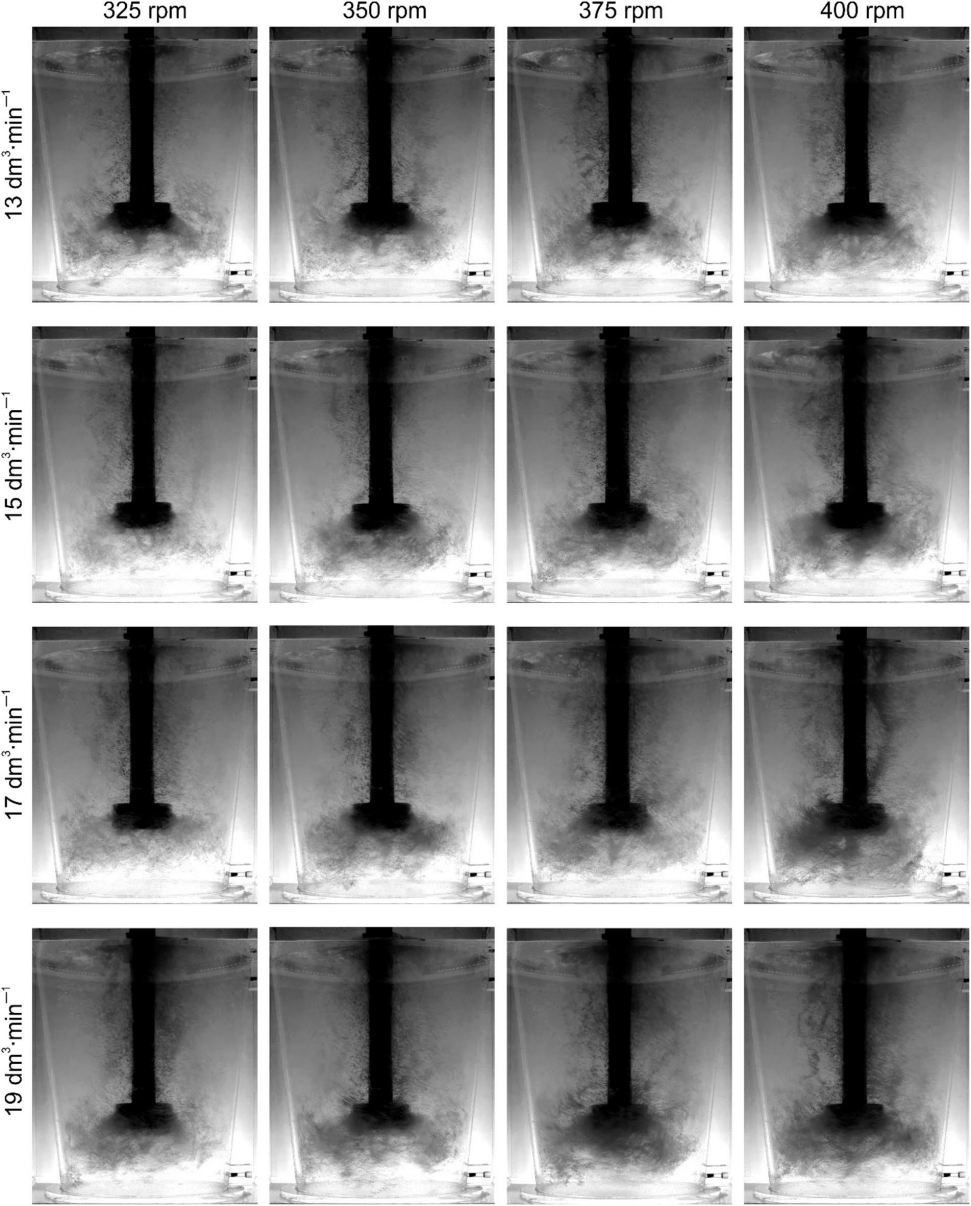
Demonstration of the nature of internal flows, behaviour and distribution of bubbles of injected argon—rotor A wear 100%.

**Figure 8:**
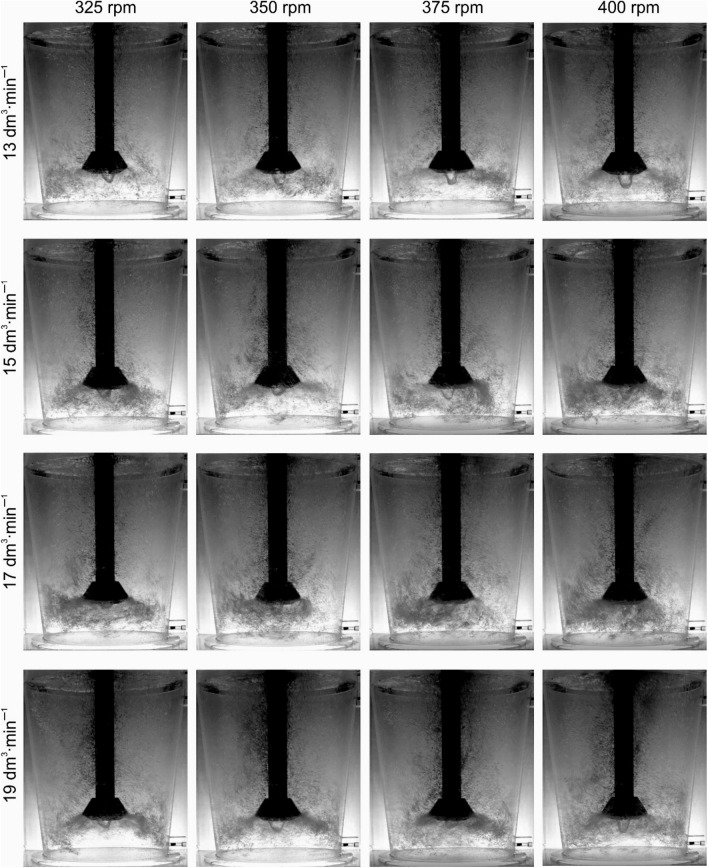
Demonstration of the nature of internal flows, behaviour and distribution of bubbles of injected argon—rotor B wear 0%.

**Figure 9:**
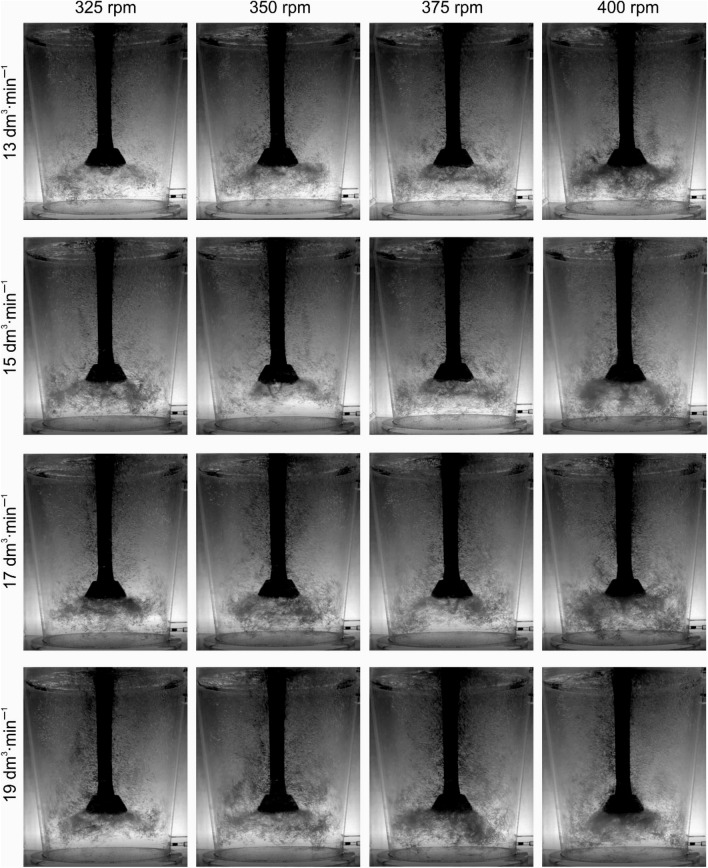
Demonstration of the nature of internal flows, behaviour and distribution of bubbles of injected argon—rotor B wear 100%.

An explanation for this unexpected phenomenon was sought in the results of the visualization studies. Figures 6, 7, 8 and 9 show examples of the results of these tests. Figure 6 shows the visualization results for the new rotor A. It can be seen that the dispersion is intimate for lower values of rotary speed and lower values of gas flow rate. With a gas flow rate of 17 dm^3^^.^min^−1^ and speeds of 375 and 400 rpm, the case of uniform dispersion can be considered. However, it should also be noted here that a funnel is formed under the impeller, which is a disadvantage as it negatively affects the efficiency of degassing due to the tendency to form gas bubbles. The smaller the gas bubbles, the more favourable the degree of dispersion in the metal bath^23,36^. Experimental studies^47^ have shown that it is important that the deformed bubble efficiently extracts energy from turbulent vortices^48^, thus contributing to the behaviour of the decay and surface concentration. Additionally, from the research of Oweis et al. shows that bubble deformability accelerates the collision of the bubble with turbulent structures^49^. Therefore, the bubble-vortex interaction largely controls the process of bubble deformation and disintegration in two-phase turbulent flow^50^. Along with the wear process, the tendency to form the funnel under the impeller decreases, resulting in a more favourable environment for the bubble-vortex interaction and as a consequence the formation of uniform dispersion—Fig. 7. This can be explained by the weakening of the impeller's influence on the liquid mixing process as a result of reducing the friction forces between the edges of the impeller and the liquid^36,38^.

In the case of rotor B, a deterioration in the degree of dispersion was observed in each of the tested research variants—Fig. 8. The conditions for the formation of this funnel in the case of rotor B—Fig. 9 change to a much lesser extent than in the case of rotor A. The reason for this is the different geometry of the rotor. According to Abreu-Lopez et al.^14^ hydrodynamic properties (i.e. velocity, pressure, gas holdup, turbulent kinetic energy fields) can be used to explain the improved performance of this impeller, as it provides a much greater pumping effect, causing a huge pressure drop below the impeller, which pulls more fluid and gas ejected radially from rotor towards the bottom of the tank. This is consistent with the assumptions of Kumareshan and Joshi^51^, they stated that in agitated tanks, the quality of the flow generated by the impeller depends mainly on the design of the impeller. As the flow exits the rotor and circulates through the tank, the average kinetic energy is converted to turbulent kinetic energy and the relative distribution at any given location depends on the design of the rotor, tank and internal components such as the baffle. As it wears, the interaction of the impeller edge with the liquid changes slightly during the process.

**Figure 10:**
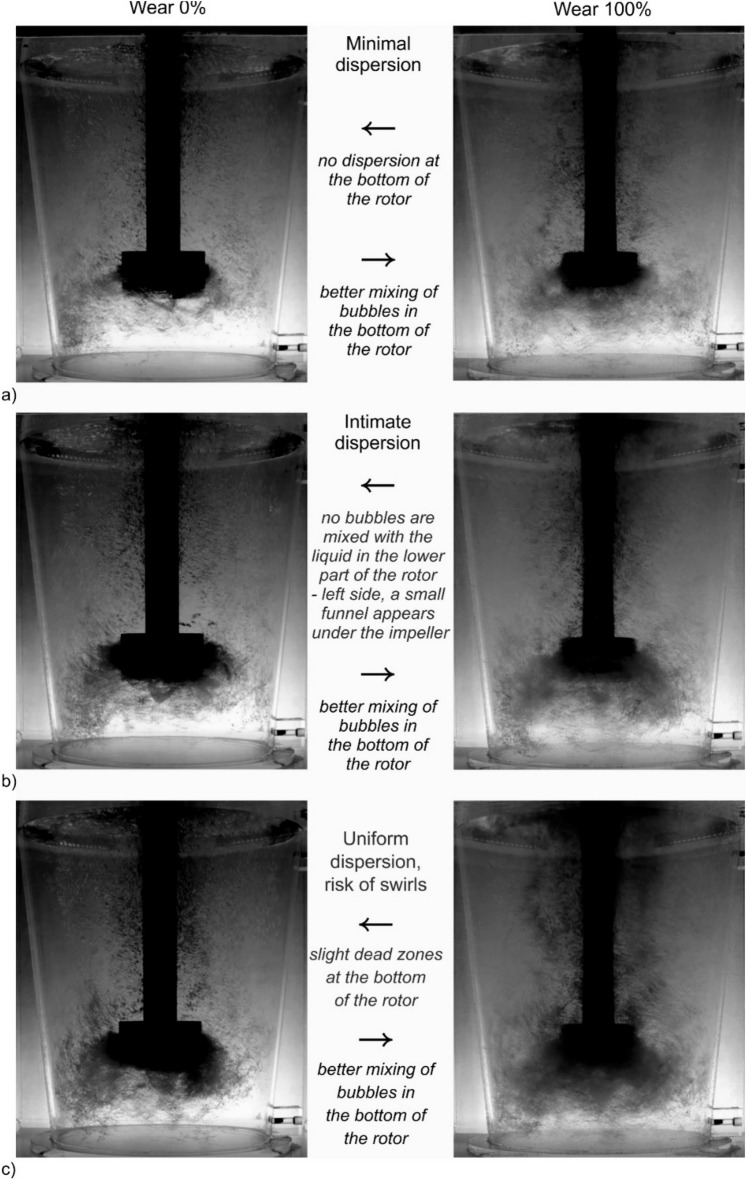
Demonstration of the nature of internal flows, behaviour and distribution of bubbles of injected argon—rotor A comparison of 0% and 100% wear: (**a**) 325 rpm; 13 dm^3^ min^−1^, (**b**) 350 rpm; 17 dm^3^ min^−1^, (**c**) 400 rpm; 19 dm^3^ min^−1^.

**Figure 11:**
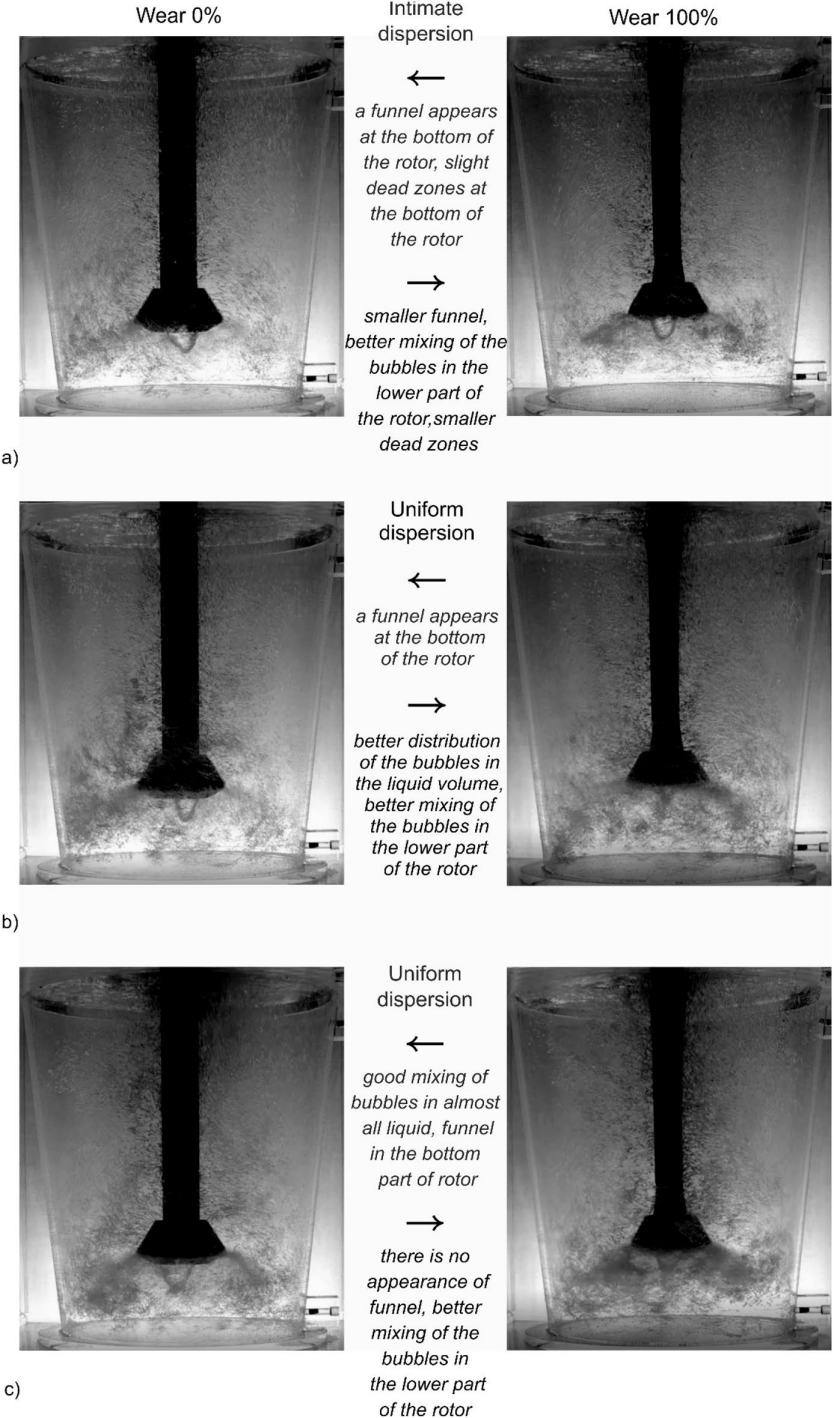
Demonstration of the nature of internal flows, behaviour and distribution of bubbles of injected argon—rotor B comparison of 0% and 100% wear: (**a**) 375 rpm; 13 dm^3^ min^−1^, (**b**) 375 rpm; 15 dm^3^ min^−1^, (**c**) 400 rpm; 19 dm^3^ min^−1^.

Figures 10 and 11 compare the most important characteristics of the dispersion type for both variants of the impeller type A and B in different phases of wear. The observed phenomena confirm the thesis presented above about the reasons for the increase in degassing efficiency with the degree of wear of the rotors.

**Figure 12:**
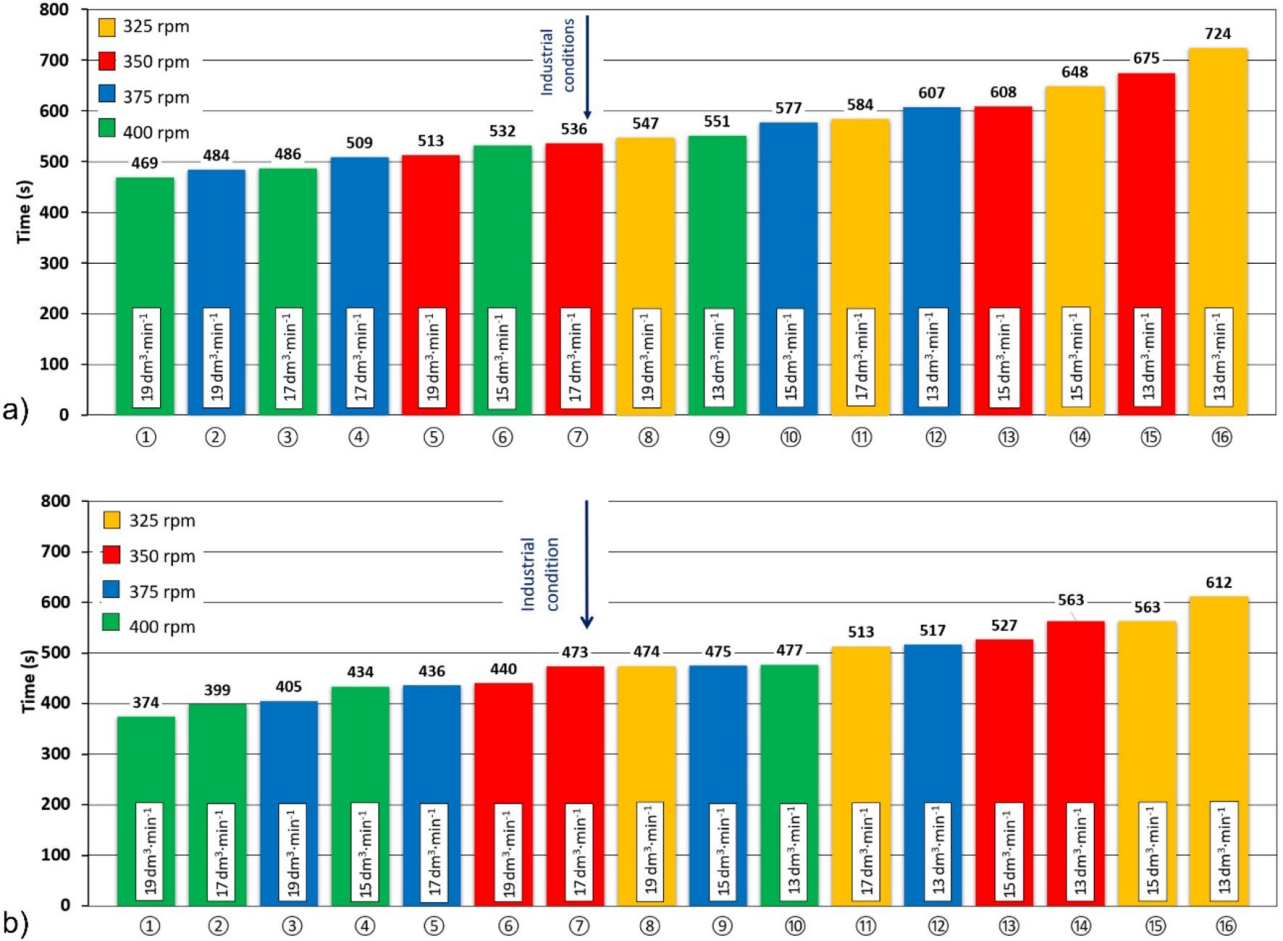
Dependence of the degassing efficiency of individual variants with impeller A with different degrees of wear depending on the time of reaching a concentration of 1 ppm of oxygen in water (degassing time): (**a**) 0% and (**b**) 100%.

Figure 12 shows the efficiency of degassing of individual variants of the experiment for a new and worn rotor A, depending on the time of reaching the oxygen concentration in water of 1 ppm (degassing time).

The graph in Fig. 12a shows that for the new impeller A, lower degassing times were achieved mainly at higher speeds and higher argon flow rates. The shortest degassing time of 469 s was obtained in variant ① with a speed of 400 rpm and an argon flow of 19 dm^3^∙min^−1^. On the other hand, variant ⑯ with 325 rpm and argon flow of 13 dm^3^∙min^−1^ reached the required oxygen concentration only after 724 s. To demonstrate the change in the efficiency of the degassing process, Fig. 12b shows the degassing times for the worn rotor A at specific rotor speeds and argon flow rates. As can be seen, in all cases the worn rotor achieved shorter degassing times than the new rotor. For example, the most efficient variant ① using a worn impeller, resulted in 95 s less degassing time than using a new impeller. Rotor wear had a clear positive effect on the process performance in the physical modelling. The use of this phenomenon in industrial conditions with the use of rotor A resulted in an increase in the efficiency of the process.

**Figure 13:**
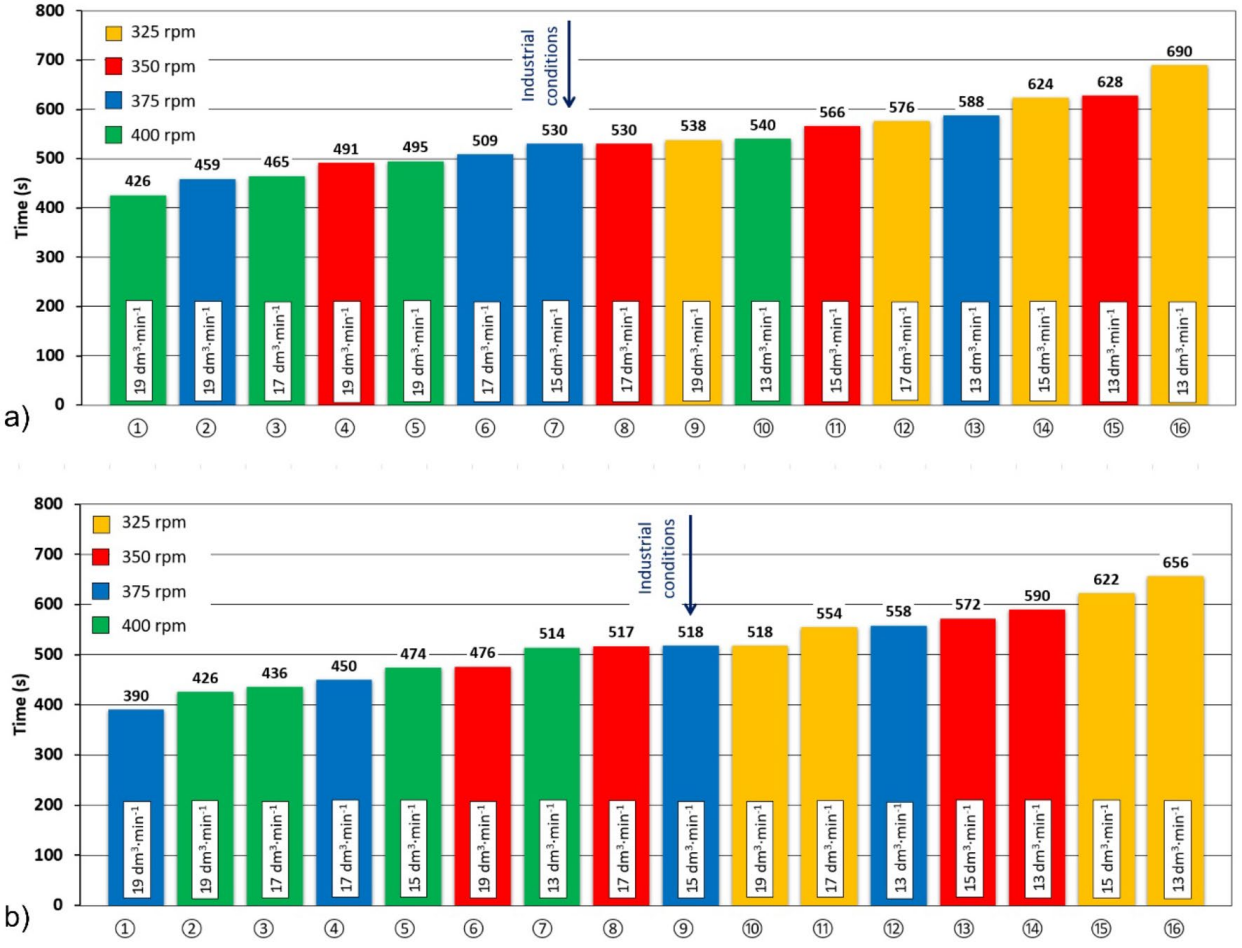
Dependence of the degassing efficiency of individual variants with impeller B with different degrees of wear depending on the time of reaching a concentration of 1 ppm of oxygen in water (degassing time): (**a**) 0% and (**b**) 100%.

Figure 13 shows the efficiency of degassing in individual variants of the experiment with the use of impeller B and with different wear depending on the duration of the degassing process until reaching a concentration of 1 ppm of oxygen in the water.

From the graph in Fig. 13 for the new impeller B, it can be seen that, as in the case of impeller A, the general trend is maintained, that lower degassing times are achieved with higher rotor speeds and higher refining gas flow rates. The lowest degassing time of 426 s was achieved in variant ① with a speed of 425 rpm and an argon flow rate of 21 dm^3^ min^−1^. On the other hand, variant ⑯ with 275 rpm and argon flow rate of 13 dm^3^ min^−1^ reached the required concentration after 690 s.

As part of the experiments, a variant used so far in industrial practice ⑦ (375 rpm, 15 dm^3^ min^−1^ Ar) was also modelled, in which the degassing time of 530 s was achieved. Figure 13a also shows the possibility of process intensification by changing one of the process parameters used. For example, when the argon flow was maintained at 15 dm^3^ min^−1^ and the rotor speed was increased to 400 rpm, the degassing time was shortened to 474 s (variant ⑤). By maintaining the speed of 375 rpm and increasing the argon flow to 17 dm^3^∙min^-1^, the degassing time was reduced to 509 s (variant ⑥). It follows from the above that the change in the rotary impeller speed has a greater impact on the tested process than the change in the argon flow rate. This is consistent with the conclusions of Gomez et al.^15^ which stated that the gas flow rate increases the gas hold up in the refining ladle, decreases vortex size and turbulence, but it decreases the stirring of the liquid because the impeller reduces the momentum transfer from the impeller to the liquid. This is also consistent with the research of Mancilla et al.^12^, who found that the impeller showed improved performance under gassed conditions, exhibiting high turbulence intensity, causing a higher rate of gas breakdown and promoting the formation of small bubbles that could be easily distributed throughout the ladle. This is also confirmed by Posadas-Navarro et al.^52^ stating that having high turbulent intensities and high energy dissipation rates guarantees a higher gas breakup rate and promotes the formation of small bubbles that distribute over the entire ladle, which leads to an improved process. Therefore, the effect may also have a positive influence on the operating conditions of the rotor, but the negative impact of increasing the rotor speed on the properties of graphite, and thus the economics of the process, should also be taken into account. The optimal selection of process parameters is therefore based on a reasonable compromise between process efficiency and its economy.

To demonstrate the change in the efficiency of the degassing process with wear of the rotor, Fig. 13b shows the degassing times for a worn rotor B, at certain rotary speeds and argon flow rates. The comparison shows that, as in the case of rotor A, there is some improvement in the efficiency of the process with the degree of rotor efficiency, but this increase is much smaller than in variant A. Therefore, the efficiency of rotor B did not show a significant dependence on the degree of rotor wear in the physical modelling.

In order to verify the results of the model tests, a metallographic analysis of the samples obtained in industrial conditions and using the analysed process parameters was carried out. Table 2 presents examples of the results of hydrogen removal tests from AlSi9Cu3(Fe) aluminium alloy in the form of a sample taken after aluminium refining for selected variants of the experiment. For all cases in Table 2, the Dichte Index was calculated to represent the hydrogen levels before and after refining. It can be seen that the Dichte Index values, i.e. the hydrogen content in the aluminium alloy, are confirmed by the model tests. In both cases, i.e. for rotors A and B, the Dichte Index values are much lower for the worn rotors than for the new rotors. This is also confirmed by the images of metallographic microsections, in the case of rotor A, but also in the case of rotor B, there is no porosity in the samples. Slightly worse results were obtained in this respect for rotor B, but the degree of porosity of the samples is much better than for the new rotors.

**Table 2:**
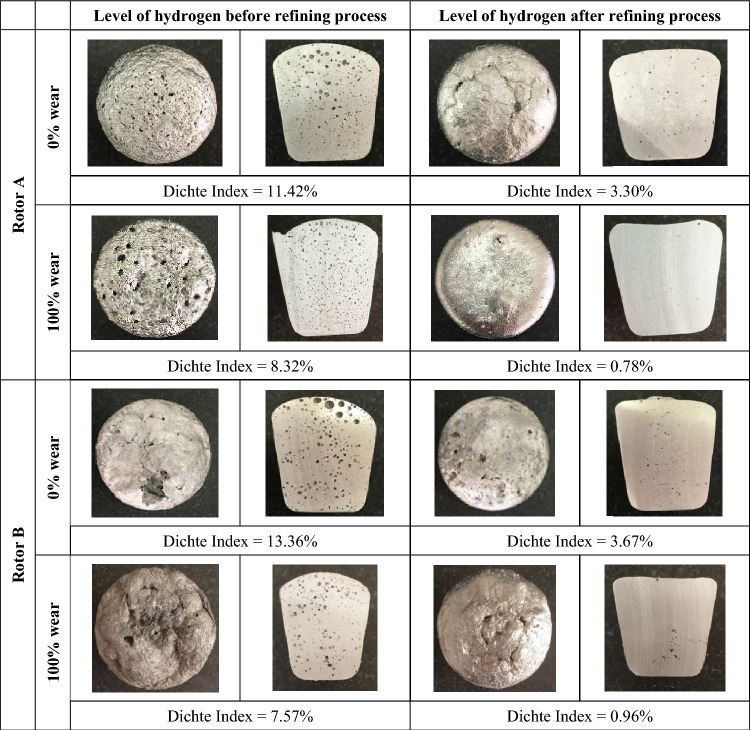
Comparison of the results of metallographic tests and the level of hydrogen content in the AlSi9Cu3(Fe) alloy before and after the refining process.

## Conclusions

On the basis of the conducted research, the following conclusions can be drawn:

- Process of aluminium refining requires the selection of appropriate process parameters that affect the efficiency of the process. The selection of these parameters is possible thanks to physical modelling—i.e. testing various rotor designs on water models. However, in order to conduct such research, it is necessary to develop an appropriate research methodology. The article develops a research methodology that has been confirmed by test results, and the results obtained are satisfactory and make it possible to test various types of rotors. The limitations in the use of this type of research result from industrial practice; the input parameters must be selected each time after consultations with the industry, so as not to lead to device failure and process disruption, while maintaining high quality of products.
- The research carried out in this article confirmed the possibility of using the tests on water models also to study the impact of the degree of rotor wear on the efficiency of the refining process. Both tested rotors are characterized by effective hydrogen removal from liquid aluminium. Generally, both model and metallographic tests show that rotor A gives better degassing results. Research has shown that rotors in the final phase of their life cycle are characterized by a better degree of hydrogen removal, which may be related to the higher content of hydrogen in new rotors.
- The two tested rotors also showed that the interaction of the rotor edge with the liquid slightly changes during the process, and its design significantly affects the efficiency of the process. As in the case of the process parameters, it was found that a similar level of degassing can be obtained by changing mainly the gas flow rate or rotary impeller speed.

Based on the research conducted, the following recommendations were made for:

- practitioner—process optimization is possible thanks to the use of physical modelling from the point of view of process effectiveness, selection of optimal process parameters, or elimination of porosity in castings,
- economic sector—process optimization is possible thanks to the use of a developed research methodology, taking into account the consumption of energy and materials (refining time, longer rotor operation and lower consumption), reducing the occurrence of low-quality castings (non-compliant with standards),
- policy makers—the use of the developed research methodology will allow you to look at the process from the ecological side, which is currently very desirable in the aspect of environmental technology.

In subsequent articles, it is planned to generalize the research results from the point of view of their significance for further industrial practice, forecast further development of the studied phenomenon with the possibility of industrial verification, and develop a mathematical model to describe the course of the refining process by blowing with argon.

## Data Availability

The datasets used and/or analysed during the current study available from the corresponding author on reasonable request.
